# Describing the linkages of the immigration, refugees and citizenship Canada permanent resident data and vital statistics death registry to Ontario’s administrative health database

**DOI:** 10.1186/s12911-016-0375-3

**Published:** 2016-10-21

**Authors:** Maria Chiu, Michael Lebenbaum, Kelvin Lam, Nelson Chong, Mahmoud Azimaee, Karey Iron, Doug Manuel, Astrid Guttmann

**Affiliations:** 1 Institute for Clinical Evaluative Sciences, G-106, 2075 Bayview Avenue, Toronto, ON M4N 3M5 Canada; 2 Canadian Institute For Health Information, 4110 Yonge Street, Suite 300, Toronto, ON M2P 2B7 Canada; 3 College of Physicians & Surgeons of Ontario, 80 College Street, Toronto, ON M5G 2E2 Canada; 4 Ottawa Hospital Research Institute, 725 Parkdale Ave, Ottawa, ON K1Y 4E9 Canada

**Keywords:** Health Administrative Data, Immigrant and refugee data, Record linkage, Vital statistics death data

## Abstract

**Background:**

Ontario, the most populous province in Canada, has a universal healthcare system that routinely collects health administrative data on its 13 million legal residents that is used for health research. Record linkage has become a vital tool for this research by enriching this data with the Immigration, Refugees and Citizenship Canada Permanent Resident (IRCC-PR) database and the Office of the Registrar General’s Vital Statistics-Death (ORG-VSD) registry. Our objectives were to estimate linkage rates and compare characteristics of individuals in the linked versus unlinked files.

**Methods:**

We used both deterministic and probabilistic linkage methods to link the IRCC-PR database (1985–2012) and ORG-VSD registry (1990–2012) to the Ontario’s Registered Persons Database. Linkage rates were estimated and standardized differences were used to assess differences in socio-demographic and other characteristics between the linked and unlinked records.

**Results:**

The overall linkage rates for the IRCC-PR database and ORG-VSD registry were 86.4 and 96.2 %, respectively. The majority (68.2 %) of the record linkages in IRCC-PR were achieved after three deterministic passes, 18.2 % were linked probabilistically, and 13.6 % were unlinked. Similarly the majority (79.8 %) of the record linkages in the ORG-VSD were linked using deterministic record linkage, 16.3 % were linked after probabilistic and manual review, and 3.9 % were unlinked. Unlinked and linked files were similar for most characteristics, such as age and marital status for IRCC-PR and sex and most causes of death for ORG-VSD. However, lower linkage rates were observed among people born in East Asia (78 %) in the IRCC-PR database and certain causes of death in the ORG-VSD registry, namely perinatal conditions (61.3 %) and congenital anomalies (81.3 %).

**Conclusions:**

The linkages of immigration and vital statistics data to existing population-based healthcare data in Ontario, Canada will enable many novel cross-sectional and longitudinal studies to be conducted. Analytic techniques to account for sub-optimal linkage rates may be required in studies of certain ethnic groups or certain causes of death among children and infants.

**Electronic supplementary material:**

The online version of this article (doi:10.1186/s12911-016-0375-3) contains supplementary material, which is available to authorized users.

## Background

Within Canada’s universal health care system, comprehensive health administrative data are routinely collected by each of the provincial governments. In Ontario, the most populous Canadian province with over 13.5 million residents, health services utilization data are captured for all legal residents with a valid health card number. Under approved ethical and legal permissions and with rigorous privacy and security policies in place, these data are held at the Institute for Clinical Evaluative Sciences (ICES), one of the largest repositories of health data in Canada. This has enabled ICES to use a consistent set of identifiers, including unique Ontario health card numbers, to link across health administrative databases, thus allowing researchers to build individual histories of patient cohorts and health care outcomes over time and across healthcare sectors (e.g. outpatient claims, emergency care, hospitalizations, long-term care). Secondary use of these linked Canadian data has enabled large programs of research to be conducted to answer numerous important health services, public health, and policy-relevant research questions [1–4].

Global migration is an increasingly common phenomenon, which combined with an ongoing global refugee crisis has resulted in North American and European countries receiving among the highest number of international migrants. Canada is already one of the most ethnically-diverse regions in the world with an annual intake of more than 250,000 new immigrants originating from over 170 different countries [5], of which Ontario receives over 95,000 of these new immigrants [6]. In fact, approximately 20 % of Ontario residents are immigrants [7]. Immigrant or refugee status is a key social determinant of health, which is strongly associated with mortality, disease onset and access to and quality of health care services [8–13]. However, information on immigration status and refugee status is not routinely collected in administrative health records. In addition, routine health administrative databases lack information on cause-specific mortality which is critical to understanding population burden of illness. ICES has recently acquired data from Immigration, Refugees and Citizenship Canada Permanent Resident (IRCC-PR, formerly Citizenship and Immigration Canada) database for more general use (in the past this has only been used for a limited number of approved projects) and the Office of the Registrar General’s Vital Statistics–Death (ORG-VSD) registry to fill in these important data gaps and to facilitate new lines of research [14, 15].

Linkage of records across databases has become an important tool for combining records that belong to the same entity across different data sources [8, 9, 16–20]. This activity consists of matching records in one database to records in another database, often a population registry, and creating a unique encoded identifier that is identical across the databases (henceforth called “record linkage”). The percentage of records in the one database that can be successfully matched to a record in the population registry is considered the “linkage rate”. This unique encoded identifier can later be used to combine these and other databases for research studies (henceforth called “joining up” databases). Record linkage is routinely used at ICES to create datasets that can be joined up for the purpose of increasing the comprehensive information available on individuals for research; however, the methods by which the record linkages are performed on Ontario health and death registry data have not been previously published. Deterministic record linkage is the simpler method that matches records if some or all identifiers (e.g. birth date, full name, health card number) are identical. When unique identifiers are not available or deterministic record linkage is not possible, probabilistic record linkage may be used to create additional matches based on probability scores that pair records belonging to the same individual. The success of record linkages is dependent on the quality of the individual data sources and identifiers as well as the accuracy of the record linkage process, which often involves manual review. The goal is therefore to reduce the number of mismatches and unlinked records and in turn to reduce the potential for systematic biases [21, 22]. There is an increasing awareness of bias that may potentially be created by excluding unlinked records from study analyses and the recently released RECORD reporting guidelines for studies using administrative health data highlight the importance of reporting of record linkage results [23]. However, there are only a limited number of studies [24, 25] that have systematically examined differences between records that have and have not been linked in large population-based data repositories.

The objectives of this paper were to describe data acquisition and the record linkage strategy used to prepare the Immigration, Refugees and Citizenship Canada Permanent Resident database and the Office of the Registrar General’s Vital Statistics–Death registry data for research; to estimate the linkage rates for each data set; and to compare the sociodemographic and other individual characteristics of the linked and unlinked populations in each database.

## Methods

### Collection of data at ICES

During the spring of 2011, IRCC and ICES partnered to develop a data sharing agreement for the disclosure of data elements of federal immigration records from IRCC to ICES. This was designed to support health services research and statistical analysis of immigrants and refugees to Ontario. The entire Ontario IRCC-PR database consisted of over 3 million records of individuals who landed in Ontario between January 1985 and December 2012. The personal identifiers included record identification number, surname, given names, date of birth, sex and landing date, as well as socio-demographic data fields, such as country of origin, last permanent residence, marital status and immigrant class. The three main immigrant classes include: economic (e.g. skilled workers, business class immigrants (investors or entrepreneurs)), family class (family reunification and sponsorship), and refugee or asylum seekers.

In a similar way, a data sharing agreement was reached between ICES and the Office of the Registrar General of Ontario to facilitate the disclosure of registered vital statistics death information to ICES. Since then, annual data updates have raised the total number of records to almost 2 million as of March 31, 2013. The personal identification fields included surname and given names, sex, postal code, dates of birth and death, in addition to details of immediate and primary cause of death using the International Classification of Diseases – Ninth (ICD-9) and Tenth (ICD-10) Revision, and place of injury and death.

The raw databases were disclosed to ICES’ eight designated data covenantors, who are authorized by the Ontario Information and Privacy Commissioner to collect data from the data partners and have access to direct personal identifiers for the purposes of conducting data record linkages at ICES.

### Record linkage

A detailed overview of the data linkage process can be found elsewhere [26, 27]. The Registered Persons Database (RPDB) represents the base population file of all legal residents in Ontario who are eligible for provincial health care coverage and captures the majority of Ontario’s 13.5 million residents. Raw data updates are provided to ICES monthly by the Ontario Ministry of Health and Long-Term Care under a specific data sharing agreement and are augmented with other administrative databases to create the final RPDB file at ICES. The RPDB file contained individual health card number, as well as personal identification information (e.g., surname, given names, sex, date of birth, earliest date of coverage, last time having contact with the health care system, and residential postal code). Records in the IRCC-PR database and ORG-VSD dataset were linked to the RPDB using the AutoMatch probabilistic record linkage program [28]. Because there was no common unique identifier between the files, the extracted personal identifiers were used for matching with the additional data standardization of surnames to augment the record linkage process by implementing the New York State Identification and Intelligence System (NYSIIS) [29] phonetic conversion. The record linkage process involved iterations of pairing each subject with the up-to-date RPDB records using a combination of last and given name variants, date of birth, sex, and in the case of the ORG-VSD, death date.

For any files of reasonable size like the administrative data, it is not feasible to compare all record pairs since the number of possible pairs is the product of the number of records on each file. For instance, if both files contain one million records, the total number of possible pairs will be one trillion. In order to optimize the scanning process of possible matched pairs, a technique called blocking was implemented. This method partitioned both files into mutually exclusive and exhaustive subsets and we looked for matches within each subset.

The RPDB database was first stratified by sex to reduce the total number of comparisons, followed by rounds of deterministic record linkage based on three blocking schemes of personal identifier variants. If an exact match could not be confirmed because of misspelled names or miscoded fields, the record linkage process continued to look for plausible matches probabilistically by subsequently utilizing a different probabilistic blocking scheme at each pass to generate both definite matches and a grey area of possible matches which were subject to the manual review process (see Additional file 1: Table S1 for details). Whether a match was considered definite or possible was based on the overall odds in favour of a true match derived from the Bayes Theorem and the user-defined threshold [30]. In order to improve accuracy of the manual process, the Statistics Canada Postal Code Conversion File was utilized to generate the corresponding city location (e.g., city of Toronto or Ottawa) from postal codes of residence to compare the geographic information between both the IRCC-PR and ORG-VSD databases against RPDB records during the review of the uncertain matched pairs. Finally, within the IRCC-PR and ORG-VSD databases, individuals who were linked to the RPDB were assigned unique ICES key numbers, which are unique identifiers derived from individual health card numbers. It is through these ICES key numbers that individual-level information is combined across administrative, clinical and survey databases to conduct research. After the completion of the record linkage process for the ORG-VSD data, duplicate death records for the same individuals were removed by retaining the match with the best record linkage quality, or comparing the date of death to the RPDB registry and keeping the record with the closest date of death. For the IRCC-PR database, records with the earliest landing date are selected.

### Statistical analysis

After record linkage was complete, identifiers (e.g., names) were removed and these anonymized datasets were used to calculate linkage rates and prevalence estimates for linked and unlinked datasets. We examined the number of records linked by deterministic and probabilistic record linkage in each step of the process, as well as the linkage rates over time. The prevalence rates of socio-demographic and geographic characteristics were calculated for the records that did and did not link to the RPDB population (i.e. where an ICES unique identifier could not be attached to the record). Given the very large sample sizes, *p*-values were not used for statistical testing; instead, prevalence estimates between the linked and unlinked samples were compared using standardized differences to assess systematic bias as suggested by Cohen [31], with 0.2, 0.5, and 0.8 representing small, moderate, and large standardized differences, respectively. Data elements of interest in the ORG-VSD data included age at death, sex, cause of death and fiscal year of death. Cause of death was categorized into broad categories of death based on ICD-9 codes. Data elements of interest in the IRCC-PR database included immigrant class, sex, marital status, and age at landing, year of entry into Ontario, as well as geographical attributes such as country of birth. The geographic attributes were grouped into 4 main world regions and 18 sub-regions according to the Standard Classification of Countries and Areas of Interest.

## Results

### IRCC permanent resident linkage rates and characteristics of linked and unlinked records

**Fig. 1:**
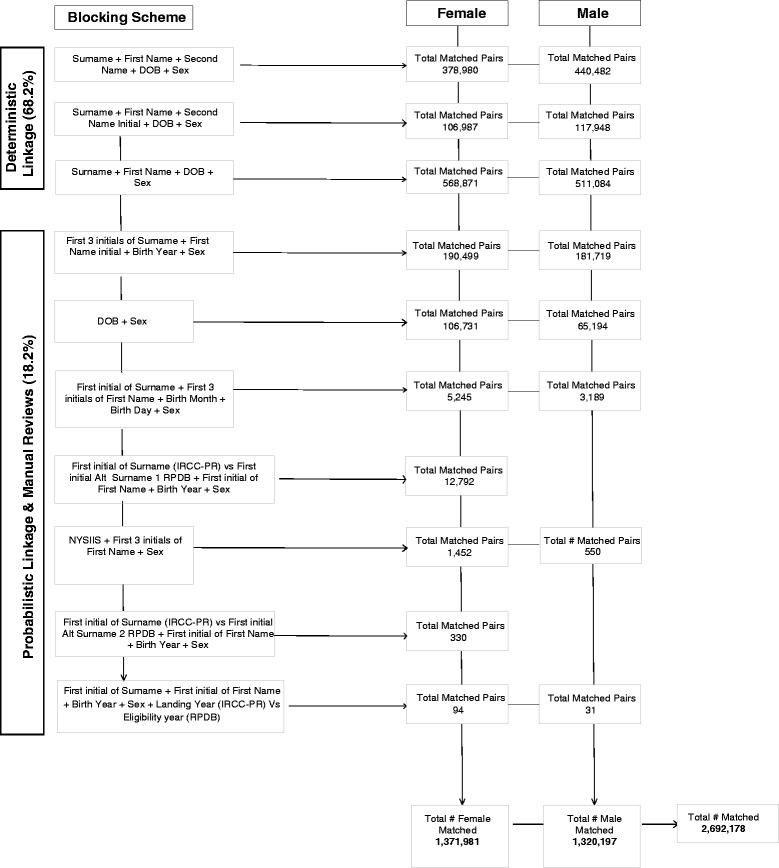
Deterministic and Probabilistic Linkage process used to link the IRCC-PR database to the RPDB. Legend. Abbreviations: IRCC-PR, Immigration, Refugees and Citizenship Canada Permanent Resident file; DOB, date of birth; RPDB, Registered Persons Database

**Fig. 2:**
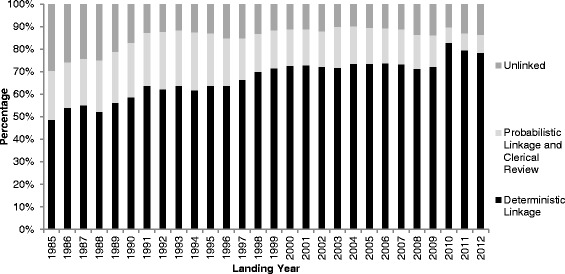
Deterministic and Probabilistic Linkage rates and percentage Unlinked for the IRCC-PR by Landing Year

There were a total of 3,117,334 immigration records captured for those who landed in Ontario between January 1, 1985 and December 31, 2012, of which 2,692,178 were linked (overall linkage rate: 86.4 %) (Table 1). The majority (68.2 %) of the record linkages in the IRCC-PR database were achieved after three deterministic passes, 18.2 % were linked probabilistically, and 13.6 % were unlinked (Fig. 1). The linkage rates gradually improved over time: from 70.5 % in 1985 to 86.4 % in 2012 (Fig. 2) (Table 1).

**Table 1:** Socio-demographic and Geographic Attributes in the IRCC-PR Database, January 1, 1985 to December 31, 2012^a^

	Linked		Unlinked		Standardized Difference	Linkage Rate (%)
	N	%	N	%		
Overall	2692178		425156		NA	86.4
Age						
0–14	562971	20.9	94066	22.1	0.03	85.7
15-–24	444631	16.5	63434	14.9	0.04	87.5
25–44	1246212	46.3	210673	49.6	0.07	85.5
45–64	333864	12.4	43401	10.2	0.07	88.5
65–84	101206	3.8	11486	2.7	0.06	89.8
85+	3293	0.1	2073	0.5	0.07	61.4
Missing	1	0	23	0	0.01	4.2
Sex						
Female	1371981	51.0	229325	53.9	0.06	85.7
Male	1320197	49.0	195365	46.0	0.06	87.1
Missing	0	0	466	0.1	0.05	0
Year of Landing						
1985–1989	279144	10.4	89577	21.1	0.30	75.7
1990–1994	544215	20.2	81689	19.2	0.03	86.9
1995–1999	475300	17.7	74667	17.6	0	86.4
2000–2004	589321	21.9	71234	16.8	0.13	89.2
2005–2012	804198	29.9	107989	25.4	0.10	88.2
Immigration Class						
Family Class	946189	35.1	108446	25.5	0.21	89.7
Economic Immigrants	1277970	47.5	270399	63.6	0.33	82.5
Refugees/Asylum Seekers	405039	15.0	36974	8.7	0.20	91.6
Other	42896	1.6	5455	1.3	0.03	88.7
No categorization	20084	0.7	3882	0.9	0.02	83.8
Marital Status						
Married/Common-Law	1364683	50.7	210753	49.6	0.02	86.6
Single	1209074	44.9	200247	47.1	0.04	85.8
Widowed/Divorced/Separated	117987	4.4	14075	3.3	0.06	89.3
Missing	434	0	81	0	0	84.3
Region of Birth						
Americas						
North America	66963	2.5	16521	3.9	0.08	80.2
Central America	53667	2.0	6402	1.5	0.04	89.3
Caribbean/Bermuda	154328	5.7	12360	2.9	0.14	92.6
South America	132972	4.9	15087	3.5	0.07	89.8
Europe						
Western Europe	31213	1.2	6961	1.6	0.04	81.8
North Europe	71056	2.6	13471	3.2	0.03	84.1
Eastern Europe	241900	9.0	23958	5.6	0.13	91.0
Southern Europe	116687	4.3	12948	3.0	0.07	90.0
Asia and Pacific						
West Central Asia and the Middle East	293208	10.9	39958	9.4	0.05	88.0
Eastern Asia	451486	16.8	127466	30.0	0.32	78.0
South East Asia	254888	9.5	37163	8.7	0.03	87.3
Southern Asia	612233	22.7	82627	19.4	0.08	88.1
Oceania	8340	0.3	1467	0.3	0.01	85.0
Africa						
Western Africa	41188	1.5	4068	1.0	0.05	91.0
Northern Africa	49382	1.8	11072	2.6	0.05	81.7
Central Africa	10155	0.4	1310	0.3	0.01	88.6
Eastern Africa	87149	3.2	9538	2.2	0.06	90.1
Southern Africa	14559	0.5	2401	0.6	0	85.8
Country not stated	804	0	378	0.1	0.02	68.0

Overall, the small standardized differences across nearly all sociodemographic factors and regions of birth indicate that the linked IRCC-PR database was largely representative of the original unlinked IRCC-PR database (Table 1). For example, we were able to successfully link 405,039 refugees/asylum seekers with a 91.6 % linkage rate. The lowest linkage rates were observed among economic immigrants (linkage rate: 82.5 %), immigrants from Eastern Asia (linkage rate: 78.0 %), those aged 85 years or over (linkage rate 61.4 %) and those who landed in the first time period, 1985–1989 (linkage rate: 75.7 %) (Table 1).

### Vital statistics–death linkage rates and characteristics of linked and unlinked records

**Fig. 3:**
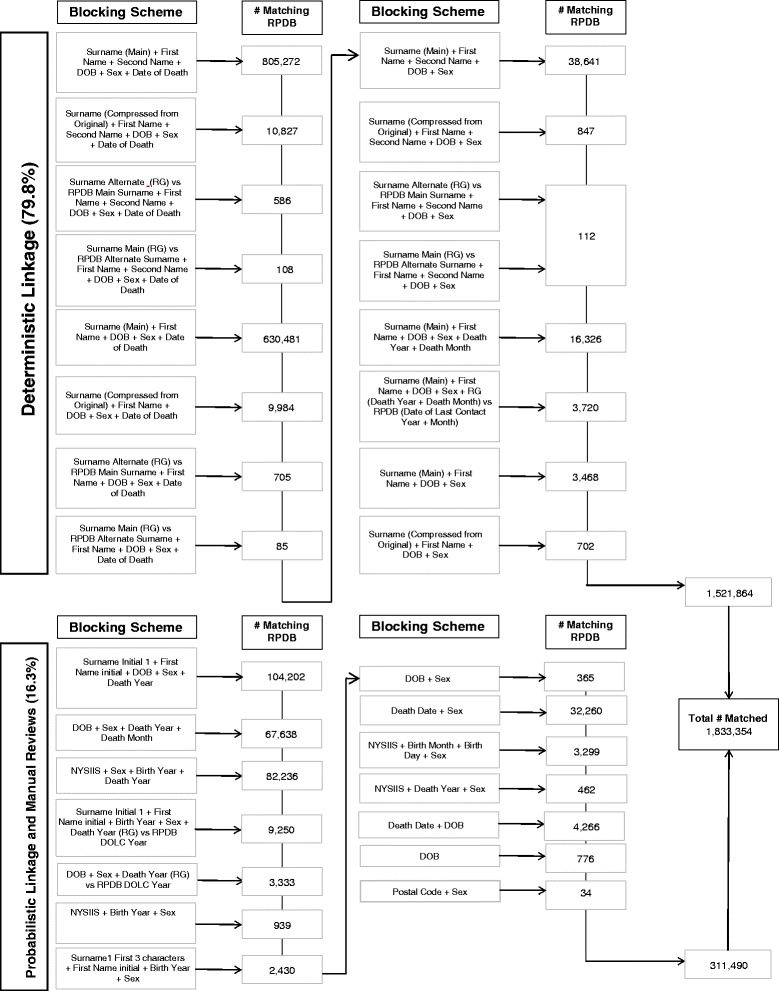
Deterministic and Probabilistic Linkage process used to link the ORG-VSD-Death to the RPDB. Legend. Abbreviations: DOB, date of birth; DOLC, date of last contact; NYSIIS, New York State Identification and Intelligence System; RG, Registrar General; RPDB, Registered Persons Database

**Fig. 4:**
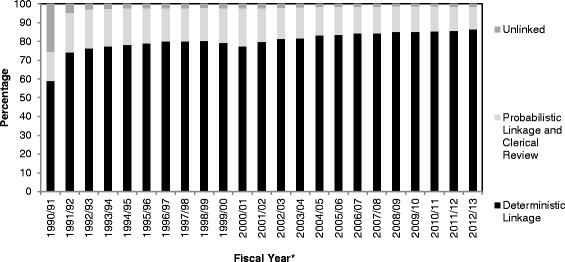
Deterministic and Probabilistic Linkage rates and percentage Unlinked for the ORG-VSD Data by Fiscal Year. Legend. ^a^Fiscal Year: April 1^st^ to March 31^st^ (e.g. Fiscal year 1990/91 is April 1, 1990 to March 31, 1991)

Between January 1^st^ 1990 to March 31, 2013, a total of 1,906,727 deaths were reported in the ORG-VSD data, of which 1,833,354 (96.2 %) were linked (Fig. 3). A total of 79.8 % were linked using deterministic record linkage, 16.3 % were linked after probabilistic linkage and manual reviews, and 3.9 % were unlinked. The linkage rates steadily improved from 74.5 % in fiscal 1990/91 to 98.6 % in 2012/13 (Fig. 4).

There were differences in the distribution of age at death, causes of death and year of death between linked and unlinked data. Individuals who died before the age of 45 years (especially those who died between ages 0 to 14 years) were less likely to be linked (Table 2). The linkage rates were generally high for most causes of death, including the two leading causes of death: diseases of the circulatory system (linkage rate: 95.9 %) and neoplasms (linkage rate: 97.1 %), which together represented 63.2 % of all deaths in the linked files. However, relatively low linkage rates were found for congenital anomalies and certain conditions originating in the perinatal period (81.3 % and 61.3 %, standardized differences: 0.16 and 0.30, respectively).

**Table 2:** Match Results in the ORG-VSD Data, January 1, 1990 to March 31, 2013

	Linked		Unlinked		Standardized Difference	Linkage Rate (%)
	N	%	N	%		
Overall	1833354		73373			96.2
Age at Death						
0–14	19483	1.1	6293	8.6	0.36	75.6
15–24	15204	0.8	1517	2.1	0.10	90.9
25–44	70023	3.8	5497	7.5	0.16	92.7
45–64	293856	16.0	14126	19.3	0.08	95.4
65–84	909667	49.6	32052	43.7	0.12	96.6
85+	525085	28.6	13787	18.8	0.23	97.4
Missing	36	0	101	0.1	0.05	26.3
Sex						
Female	906234	49.4	31261	42.6	0.14	96.7
Male	927110	50.6	42077	57.3	0.14	95.7
Missing	10	0	35	0	0.03	22.2
Cause of death (ICD-9)^a^						
Infectious and Parasitic Diseases (001–139)	32456	1.8	1222	1.7	0.01	96.4
Neoplasms (140–239)	536603	29.3	15780	21.5	0.18	97.1
Endocrine, Nutritional, and Metabolic Diseases and Immunity Disorders (240–279)	74205	4.0	2352	3.2	0.05	96.9
Diseases of the Blood and Blood-Forming Organs (280–289)	5940	0.3	248	0.3	0	96.0
Mental Disorders (290–319)	62774	3.4	1301	1.8	0.10	98.0
Diseases of the Nervous System (320–359)	70297	3.8	1676	2.3	0.09	97.7
Diseases of the Sense Organs (360–389)	100	0	6	0	0	94.3
Diseases of the Circulatory System (390–459)	621713	33.9	26744	36.4	0.05	95.9
Diseases of the Respiratory System (460–519)	153350	8.4	5466	7.4	0.03	96.6
Diseases of the Digestive System (520–579)	72477	4	2644	3.6	0.02	96.5
Diseases of the Genitourinary System (580–629)	35042	1.9	968	1.3	0.05	97.3
Complications of Pregnancy, Childbirth, and the Puerperium (630–679)	134	0	13	0	0.01	91.2
Diseases of the Skin and Subcutaneous Tissue (680–709)	3008	0.2	97	0.1	0.01	96.9
Diseases of the Musculoskeletal System and Connective Tissue (710–739)	10094	0.6	246	0.3	0.03	97.6
Congenital Anomalies (740–759)	7246	0.4	1671	2.3	0.16	81.3
Certain Conditions Originating in the Perinatal Period (760–779)	5846	0.3	3688	5.0	0.30	61.3
Symptoms, Signs, and Ill-Defined Conditions (780–799)	20624	1.1	1942	2.6	0.11	91.4
External Causes of Injury and Poisoning (E000-E999)	105662	5.8	7014	9.6	0.14	93.8
Missing^b^	15783	0.9	295	0.4	0.06	98.2
Fiscal Year of Death^c^						
1990–1994^d^	346805	18.9	45885	62.5	0.99	88.3
1995–1999	392282	21.4	9234	12.6	0.24	97.7
2000–2004	406196	22.2	8406	11.5	0.29	98.0
2005–2009	428974	23.4	6207	8.5	0.42	98.6
2010–2012	259097	14.1	3616	4.9	0.32	98.6
Missing	0	0	25	0	0.03	0

^a^ICD-9, International Classification of Diseases – Ninth Revision

^b^cause of death is only available until December 31, 2012. Only 1088 records among the linked file and 113 records among the unlinked file were missing prior to January 1st 2013

^c^Fiscal years: April 1 to March 31. Death date is available until March 31, 2013

^d^First period 1990–1994 started in January 1, 1990

## Discussions

In this study, we described the techniques used to link the IRCC-PR and the ORG-VSD databases to the population-based dataset containing all registered persons in Ontario. We observed an improvement in the overall linkage rates of population-based immigration and death records data over time. The overall linkage rates for the IRCC-PR and ORG-VSD databases were high at 86.4 and 96.2 %, respectively. However, a comparison of the linked and unlinked files suggests that a few systematic biases may have been introduced when linking the data. The lowest linkage rates in the IRCC-PR database were found among immigrants born in East Asia, meanwhile the category of deaths in the ORG-VSD data with the lowest linkage rates was deaths to due conditions arising in the perinatal period.

East Asian immigrants (e.g., from China, Japan, South Korea, etc.) had one of the lowest linkage rates and were the leading source of unlinked cases. This is likely due to common East Asian surnames, such as Lee, Li, and Kim. Previous research developing an algorithm based on surnames to identify Chinese and South Asians within ICES data also demonstrated high specificity, but low sensitivity among Chinese individuals due to these common surnames [32]. Shorter surnames, most typical of ethnic Chinese surnames, may be particularly vulnerable to lower linkage rates, because a single discrepancy in a two- or three-character string can trigger higher negative agreement weights, and hence reduce the likelihood of a positive match. Further investigation is needed to determine whether the lower linkage rates among East Asian immigrants may affect the findings of research studies and if more advanced record linkage techniques can improve these linkage rates.

The overall linkage rate for the Ontario ORG-VSD data was comparable to a previous study where deterministic record linkage was applied to link vital statistics data to the population registry in Alberta, another province in Canada [33]. It is important to note that we were able to achieve similarly high linkage rates with a 90-fold greater number of records and with a 6-times longer data coverage period than the earlier study. The lowest linkage rates among the age groups in the ORG-VSD was found in the youngest (0 to 14 year) age group, a finding consistent with the earlier study in Alberta [33]. The low linkage rates in this youngest age group may in part be explained by infant deaths occurring during the newborn hospitalization (either stillbirths or early neonatal deaths). Under the Provincial and territorial Vital Statistics Acts [34], the registration of stillbirths is a legal requirement in each Canadian province and territory, but only sex and date of birth or death are required for the registration, thus decreasing the likelihood of linkage. Furthermore, some of these babies with early neonatal deaths may not be issued health care numbers and as such would not be included in the Registered Persons Database. This has important implications for perinatal research using these linked data.

Low linkage rates and errors in record linkage can lead to biased results [35]; therefore, identifying the source of error and mitigating it is important for the purpose of using linked data for population health research. Previous studies have investigated the correction of record linkage error. For example, Hagger-Johnson et al. [36], who described the scenario of falsely matching infant and preterm records, suggested removing clinically implausible scenarios of those affected as early as during the data cleaning stage. There are several ways to estimate bias as a result of record linkage error [37]. In particular, a gold-standard dataset where true matches have been identified can be compared to the linked pairs. Sensitivity analysis can also be carried out to provide the range of plausible results, especially in the case where record linkage was determined by subjective clerical review. Harron et al. also used other methods to evaluate the impact of record linkage error on estimations including the highest-weight classification method which links records with the highest probabilistic match weights (probability of agreeing on identifiers given their match status) above a specified threshold and prior-informed imputation using match probabilities (probability of a match given they agree on a set of identifiers) and only transfers variables of interest rather than the whole record to the primary file [35, 37]. As recommended by the RECORD statement, health researchers should report on the estimated rates of deterministic, probabilistic and manual linkage and, if available, researchers should also consider presenting information about the unlinked data, so that readers are able to determine how the linked and unlinked data affect results [23]. In addition, we recommend disclosing the manual review process used to determine resolution of possible matches and to ensure that steps are taken to minimize the number of false positive and false negative matches.

The record linkages of vital statistics and immigration data to the RPDB represent significant advances in ICES’ data holdings that will enable many novel population health and health services research studies to be conducted. Record linkage of ORG-VSD registry to ICES data holdings allows for longitudinal follow-up of causes of death across the entire Ontario population which, at over 13.5 million individuals, is larger than several European countries with administrative databases that are used for research, such as Sweden, Denmark and Finland. This enables the study of relatively rare causes of death, such as suicide [14]. By joining up the linked IRCC-PR database to other databases, immigrants can be followed longitudinally to study patterns of health services utilization across most healthcare sectors, including hospital, outpatient, emergency, and long-term care. In addition, the IRCC-PR database can be merged with validated ICES disease cohorts to study the prevalence and incidence of diabetes [38], asthma [39], congestive heart failure [40] and numerous other conditions. Furthermore, given the growing global refugee crisis and influx of refugees to developed countries, linkages of data such as the IRCC-PR database to health care administrative databases will enable research to be conducted that can inform the delivery of health care services to and the assessment of health outcomes among Syrian and other refugee populations. Elements of the IRCC-PR database, have already been joined up with other population health data to create and validate an algorithm for ethnicity [41], another important social determinant of health that is not adequately captured in administrative databases.

The databases involved in these record linkages are not without limitations. First, IRCC-PR database are only available since 1985, therefore we are not able to study immigrants who have lived in Canada for more than 27 years as of the 2012 data update. Earlier studies, however, have shown that many health factors and behaviors of immigrants converge with those of non-immigrants after immigrants have lived in Canada for at least 15 years [42] or 20 years [43]. Nevertheless, users of the data need to acknowledge this limitation of the IRCC-PR database. Second, the current IRCC-PR database at ICES only captures immigrants who migrated directly to Ontario and not those who first landed in a different province. A general limitation of IRCC-PR database and ICES data is that we currently do not have records of emigration. Researchers have addressed this limitation by examining periods of no contact with the health care system in populations that ought to be using the health care system and by examining the end of eligibility of health care coverage [44].

## Conclusions

In conclusion, the overall high linkage rates for both immigration and death records suggest that the combined strategy of deterministic and probabilistic record linkage with manual review using personal identifiers can greatly enhance the ability to do research on large population-wide databases. However, we have shown that there may be important differences in unlinked and linked populations which need to be acknowledged when using these data for research. Specifically, analytic techniques to account for sub-optimal linkage rates may be required in studies of certain ethnic groups or certain causes of death among children and infants. The record linkage approach we describe in this paper is relevant to other jurisdictions with similar administrative data sources and provides an opportunity for health and non-health related information to be brought together to provide a comprehensive view of individuals’ life histories.

## Additional file

Additional file 1: Table S1. Manual review process to determine resolution of possible matches. Lists the matching criteria used to determine possible matches. (DOCX 15 kb)

## Abbreviations

ICD-10: International Classification of Diseases – Tenth Revision
ICD-9: International Classification of Diseases – Ninth Revision
ICES: Institute for Clinical Evaluative Sciences
IRCC-PR: Immigration, Refugees and Citizenship Canada Permanent Resident database
NYSIIS: New York State Identification and Intelligence System
ORG-VSD: Office of the Registrar General’s Vital Statistics-Death registry
RPDB: Registered Persons Database
