# Supplementary material for: Describing the linkages of the immigration, refugees and citizenship Canada permanent resident data and vital statistics death registry to Ontario’s administrative health database
Source: BMC Med Inform Decis Mak. 2016 Oct 21;16:135. doi: 10.1186/s12911-016-0375-3 (PMC5073414; doi:10.1186/s12911-016-0375-3)
Supplement: Additional file 1: Table S1. — Manual review process to determine resolution of possible matches. Lists the matching criteria used to determine possible matches. (DOCX 15 kb) [file 12911_2016_375_MOESM1_ESM.docx]

**Supplementary Table 1.** Manual review process to determine resolution of possible matches

######

| **Rule** | **Matching Criteria** | **Verdict** |
| --- | --- | --- |
| 1 | Agreement on surname, first name, second name, date of birth | Match |
| 2 | Agreement on surname, first name, second name, transposition of birth month and day | Match |
| 3 | Agreement on surname, date of birth, transposition of first name and second name | Match |
| 4 | Agreement on surname, date of birth, first name nickname variant | Match |
| 5 | Agreement on surname, first name initial and second name initial, date of birth, landing date and Ontario Health Insurance Plan (OHIP) eligibility date 90 days apart. | Match |
| 6 | Agreement on uncommon surname, partial first name, date of birth, landing date and OHIP eligibility date 90 days apart | Match |
| 7 | Agreement on surname, first name initial, disagreement on date of birth | No match |
| 8 | Agreement on surname, first name but disagreement on second name initial, date of birth | No match |
| 9 | Agreement on surname, second name initial but full disagreement on first name, date of birth | No match |
| 10 | Agreement on surname, date of birth, first name absent | No match |
